# The impact of exercise intervention for patients undergoing hemodialysis on fatigue and quality of life: A protocol for systematic review and meta-analysis

**DOI:** 10.1097/MD.0000000000021394

**Published:** 2020-07-17

**Authors:** Fan Zhang, Yan Bai, Xing Zhao, Liuyan Huang, Ying Zhang, Huachun Zhang

**Affiliations:** a Department of Nephrology; b Shanghai University of Traditional Chinese Medicine; c Department of Nursing, Longhua Hospital Shanghai University of Traditional Chinese Medicine, Shanghai, China.

**Keywords:** hemodialysis, exercise, systematic review, fatigue, health-related quality of life

## Abstract

**Background:**

This study aims to determine the therapeutic efficacy of exercise interventions for patients undergoing hemodialysis (HD) on fatigue and health-related quality of life (HRQoL).

**Methods:**

This review will only include randomized controlled trials (RCTs). The search strategy will be performed in 4 English databases, 4 Chinese databases, Clinical Trials.gov, and the Chinese Clinical Trial Registry. All English or Chinese RCTs, published from inception to May 31, 2020, will be sought. Two reviewers will screen, select studies, extract data, and assess quality independently. Primary outcome is fatigue assessed by questionnaire. The methodological quality including the risk of bias of the included studies will be evaluated using the Physiotherapy Evidence Database scale. Stata 12.0 software will be used for heterogeneity assessment, generating funnel-plots, data synthesis, subgroup analysis, and sensitivity analysis.

**Results:**

We will provide some more practical and targeted results investigating the effect of exercise interventions for patients undergoing HD on fatigue and HRQoL in the current meta-analysis, and point out the main limitation of previous studies.

**Conclusion:**

The study will provide recent evidence for evaluating the therapeutic efficacy of exercise interventions for patients undergoing HD on fatigue and HRQoL.

**Registration number:**

INPLASY202050071 (DOI: 10.37766/inplasy2020.5.0071)

## 1 Introduction

### 1.1 Description of the condition

Globally, end-stage kidney disease (ESKD) is one of the leading causes of death and disability, and the number of patients is increasing steadily.^[1]^ Hemodialysis (HD) is a life-sustaining treatment for patients with end-stage renal disease (ESRD).^[2]^ In China, the prevalence of HD was 402.18 and the corresponding number of HD was approximately 553,000.^[3]^ Although ongoing technical improvement in HD has ameliorated the long-term prognosis of ESRD patients, patients still suffer a complex array of clinical symptoms.^[4]^

Fatigue is one of the most common symptoms and studies suggested a prevalence ranging from 60% to 97% in HD patients.^[5]^ It is a distressing and debilitating symptom that affects patients’ daily activities and leads to highly burdensome, impaired functioning, and health-related quality of life (HRQoL).^[6]^ Moreover, fatigue is associated with increasing risk for cardiovascular morbidity and mortality.^[5,7]^ It can be extremely weakening and intrusive both mentally and physically, which makes patients undergoing HD often experience limitations at work, in social interaction, and physical activity.^[8,9]^ The high prevalence and severe impact of fatigue on overall health and well-being may explain why fatigue is one of the top-priority issues for HD patients and health providers.^[10]^ To reiterate the universality of fatigue symptoms, Standardized Outcomes in Nephrology-Hemodialysis (SONG-HD) Consensus Workshop has established it as a critically important outcome to be reported in clinical studies in HD patients.^[11]^ Despite this, fatigue remains challenging to manage and under-recognized.

### 1.2 Description of intervention

The term exercise is defined as “planned, structured, repetitive, and purposive in the sense that improvement or maintenance of one or more components of physical fitness.”^[12]^ Epidemiological reports suggesting insufficient exercise amount is the leading risk factor for premature mortality in HD patients.^[13]^ Current Chinese guideline recommends that exercise interventions should be a component of integrated management.^[14]^ Also, trials assessing exercise interventions for fatigue have been published and add to the evidence base of the association of exercise and fatigue in HD patients.^[15]^ However, on one side, some patients, caregivers, and health providers considered that exercise would aggravate fatigue symptoms,^[16]^ on the other hand, most patients prefer to a sedentary lifestyle at home because they may experience post-dialysis fatigue providing a barrier to participating exercise.^[17]^ Therefore, a systematic review and meta-analysis will be performed to determine the therapeutic efficacy of exercise interventions for patients undergoing HD on fatigue and HRQoL, to provide further evidence for health providers and patients.

### 1.3 Objective of this study

The aim of this study was to conduct a systematic review and meta-analysis determining the therapeutic efficacy of exercise interventions for patients undergoing HD on fatigue and HRQoL.

## 2 Methods

The protocol was registered on the International Platform of Registered Systematic Review and Meta-analysis Protocols (INPLASY202050071). The preferred reporting items for systematic review and meta-analysis protocols (PRISMA) will serve as guidelines for reporting present review protocol and subsequent formal paper.

### 2.1 Inclusion criteria for study selection

#### 2.1.1 Types of studies

We will only include randomized controlled trials (RCTs), quasi-RCTs, non-RCTs, and other types of studies will be excluded.

#### 2.1.2 Types of participants

Patients with ESRD undergoing HD were diagnosed according to Kidney Disease Improving Global Outcomes (KDIGO)^[18]^ were included, regardless of age, sex, race, region, education, and type of dialysis.

#### 2.1.3 Types of interventions

The patients will receive exercise interventions, including aerobic exercise, resistance training, or combined, and balance training, flexibility training. Considering the diversity of exercise, we will include all types of exercise interventions without any restrictions on frequency, intensity, duration, and mode. Comparisons will include: placebo control, sham exercise, and stretch training; exercise interventions plus one treatment compares with the same treatment; exercise interventions compares to drug therapy.

#### 2.1.4 Types of outcome assessments

##### 2.1.4.1 Primary outcomes

Fatigue was measured subjectively by questionnaires such as fatigue-specific scales, for example, the Functional Assessment of Chronic Illness Therapy-Fatigue (FACIT-F), Piper Fatigue Scale (PFS), Brief Fatigue Inventory (BFI), Chalder Fatigue Scale (CFQ), or fatigue subscale as part of a measure assessing a broader construct, for example, SF-36 or Kidney Disease Quality of Life Instrument, or visual analogue scale. We will consider all patient-reported outcome measures for fatigue given the lack of validation work conducted in the dialysis population.

##### 2.1.4.2 Secondary outcomes

Secondary outcomes mainly involve HRQoL throughout the follow-up period.

#### 2.1.5 Search strategy

We will search Pubmed, Cochrane Central Register of Controlled Trials (CENTRAL), Web of Science, Embase as well as four Chinese databases, namely China National Knowledge Infrastructure (CNKI), Wanfang, Chinese Biomedical Literature Database (CBM), and Chinese Science and Technology Journal Database (VIP). All the English and Chinese literature published from inception to May 31, 2020 will be retrieved. In addition, we will also undertake a targeted gray literature search on Clinical Trials.gov and the Chinese Clinical Trial Registry to gain unpublished or in-progress trials or completed but prepared for publication. Meanwhile, the reference list of previous clinical studies and reviews will be searched as supplementary sources. The search strategy for CENTRAL is shown in Table 1, and similar strategies will be applied for other electronic databases.

**Table 1:**
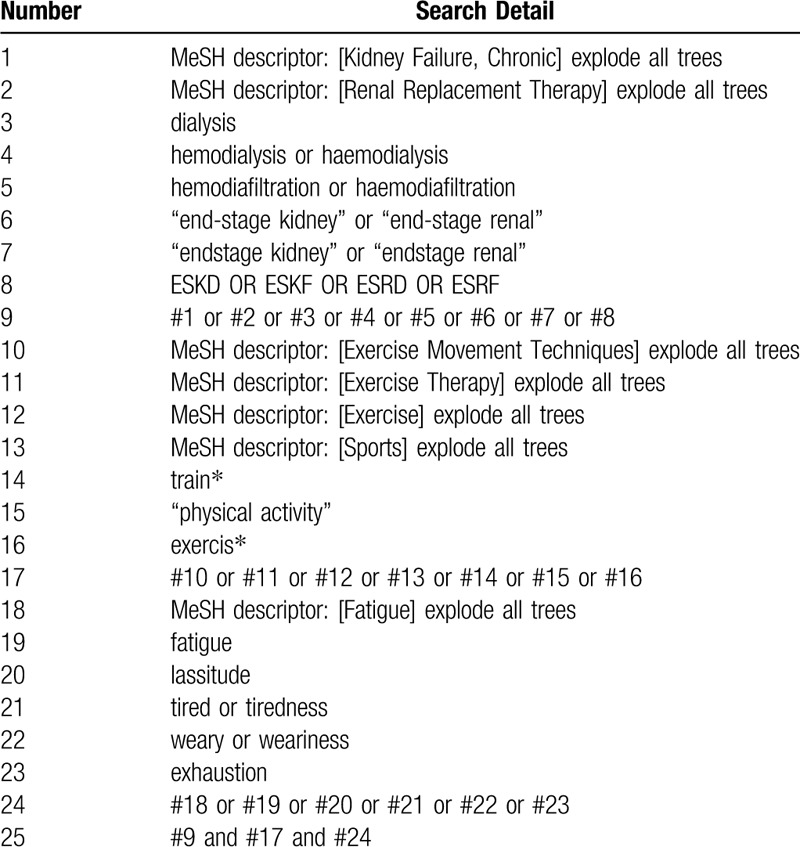
Search strategy for CENTRAL.

### 2.2 Data collection and analysis

#### 2.2.1 Selection of studies

**Figure 1:**
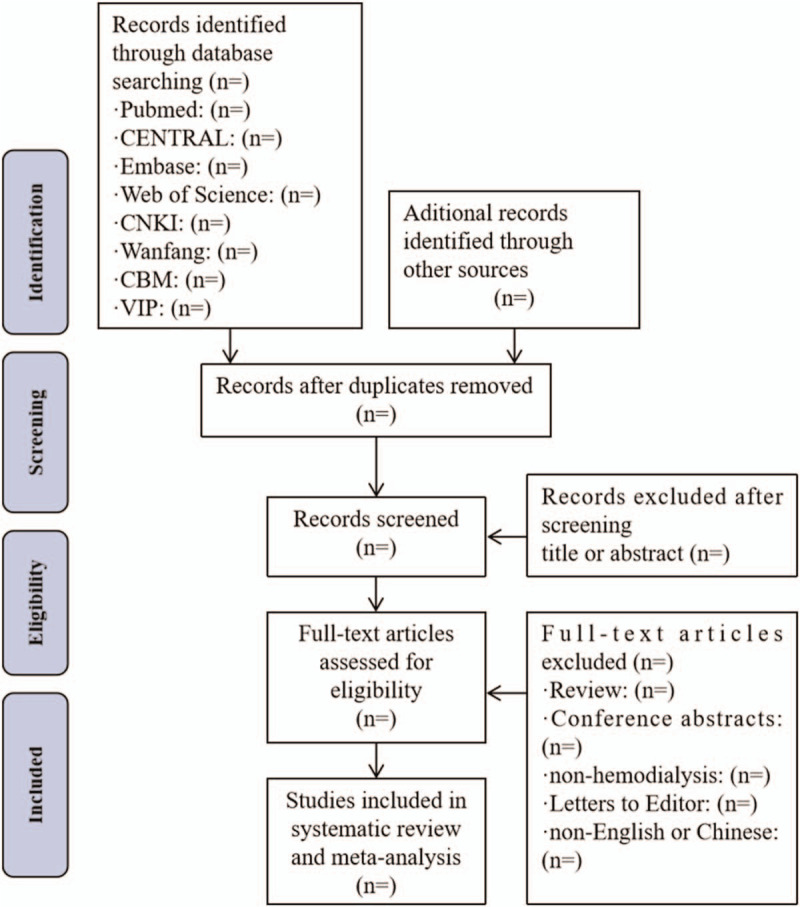
Flow diagram of study selection. CBM = Chinese BioMedical Literature Database, CENTRAL = Cochrane Central Register of Controlled Trials, CNKI = Chinese National Knowledge Infrastructure, VIP = Chinese Science and Technology Periodical Database.

Two independent reviewers (YB and XZ) will develop the literature search and screening the titles and abstracts to the inclusion criteria to exclude irrelevant studies. If necessary, the full text will be read for further assessment. The discrepancies in the process will be discussed and solved with a third author (HCZ). For unclear data, we will contact the first author to determine whether to include this literature. The detail of the study selection will be presented in a PRISMA flow diagram (Fig. 1).

#### 2.2.2 Data and information extraction

A detail data and information extraction form will be made by following: basic information (first author, publication year, country); participants’ characteristics (sample size, average age, sex ratio, dialysis vintage); interventions (exercise frequency, intensity, type, time, and duration); comparisons (control mode); outcomes (assessment tool, detail of results, adverse events).

#### 2.2.3 Dealing with missing data

If the data is incomplete or missing, we will contact the author by E-mail for information. When impossible, we will perform a sensitivity analysis to determine the impact of included studies in the overall assessment of results.

#### 2.2.4 Appraisal of study quality

Physiotherapy Evidence Database (PEDro) scale^[19]^ will be used to evaluate 11 domains of quality of each included literature by 2 independent reviewers (YB and XZ). Each item was scored with a maximum score of 10 (criterion 1 is not scored), including random allocation; allocation concealed; baseline similarity; subject blinding; therapist blinding; assessor blinding; adequate follow-up; intention-to-treat analysis; between-group comparisons; point measures and variability. Any divergences will be resolved through discussion and consultation with a third author (HCZ).

#### 2.2.5 Assessment of reporting bias

If possible, funnel plots will be used to assess for the potential existence of small study bias. Egger test and Begger analysis will be used to assess the reporting bias if necessary.

#### 2.2.6 Assessment of heterogeneity

We will quantify statistical heterogeneity using the *I*^2^ statistic to assess heterogeneity in included studies. The degree of heterogeneity is based on: *I*^2^ = 0%∼40%, might not be important; *I*^2^ = 30%∼60%, may represent moderate heterogeneity; *I*^2^ = 50%∼90%, may represent substantial heterogeneity; *I*^2^ = 75%∼100%, considerable heterogeneity.

#### 2.2.7 Measure of treatment effect

We used Stata 12.0 for meta-analysis to synthesize included data. For dichotomous variables, such as adverse events, we will calculate risk ratio with 95% confidence interval (CI). For continuous variables, mean difference (MD) or standard MD with 95% CI will be calculated. When *I*^2^ < 50%, the fixed-effect model will be selected to calculate the MD, otherwise use a random-effect model. Where the included literatures are clinically heterogeneous we will perform a narrative synthesis.

#### 2.2.8 Subgroup analysis

If there is significant heterogeneity among the outcomes, we will perform a subgroup analysis: the types of exercise interventions (resistance training vs aerobic exercise); duration of treatment (<8 weeks vs ≥8 weeks); dialysis vintage (< 5 years vs ≥ 5 years).

#### 2.2.9 Sensitivity analysis

We will conduct a sensitivity analysis by excluding merged studies one by one and observe whether the synthesis result changes significantly. If yes, the removing study may influence the overall synthesized result, so, we will reassess it and decide cautiously whether to merge it.

#### 2.2.10 Quality of evidence

We will use the Grading of Recommendations, Assessment, Development, and Evaluation to evaluate the quality of these outcomes. The outcomes will be divided into very low, low, moderate, or high.^[20]^

#### 2.2.11 Ethics and dissemination

The systematic review does not require ethical approval, because it is not individual data that is used. The results will provide evidence for the efficacy of exercise intervention in the patients undergoing HD, which will also have an importance for clinical practice and research.

## 3 Discussion

Fatigue is a feeling of exhaustion, tiredness, weariness, or lack of energy,^[21]^ which is common and potentially debilitating, and often unrecognized in HD patients.^[22]^ Although numerous studies on dialysis-related fatigue have been reported and the mechanisms behind the development of fatigue are relatively well understood,^[23,24]^ there is a lack of high-quality evidence on exercise interventions in the management of fatigue in HD patients. A recently systematic review states that exercise interventions probably have a positive effect on fatigue in adults with chronic fatigue syndrome compared to usual care or passive therapies.^[25]^ However, how strong the effects of exercise are for HD patients is still unclear. We hope to provide more practical and targeted results to identify the therapeutic efficacy of exercise intervention for HD patients on fatigue and HRQoL in the current systematic review and meta-analysis.

The strength of this systematic review and meta-analysis will include: search a comprehensive range of databases, including Chinese and English databases, more rigorous and detailed concerning quality assessment and data extraction. The limitation is that we will only consider the literature written in Chinese and English, which may omit some relevant articles published in other languages, and there may be a large heterogeneity, which may bias the results.

## Author contributions

FZ developed the search strategy and drafted the protocol, and revised by LYH and YZ; YB and XZ independently worked on study selection, quality assessment, data extraction; YZ worked on data synthesis; HCZ resolved any divergences.
